# eCOMPAGT – efficient Combination and Management of Phenotypes and Genotypes for Genetic Epidemiology

**DOI:** 10.1186/1471-2105-10-139

**Published:** 2009-05-11

**Authors:** Sebastian Schönherr, Hansi Weißensteiner, Stefan Coassin, Günther Specht, Florian Kronenberg, Anita Brandstätter

**Affiliations:** 1 Department of Database and Information Systems, Institute of Computer Science, University of Innsbruck, Innsbruck, Austria; 2 Division of Genetic Epidemiology, Department of Medical Genetics, Molecular and Clinical Pharmacology, Innsbruck Medical University, Innsbruck, Austria

## Abstract

**Background:**

High-throughput genotyping and phenotyping projects of large epidemiological study populations require sophisticated laboratory information management systems. Most epidemiological studies include subject-related personal information, which needs to be handled with care by following data privacy protection guidelines. In addition, genotyping core facilities handling cooperative projects require a straightforward solution to monitor the status and financial resources of the different projects.

**Description:**

We developed a database system for an efficient combination and management of phenotypes and genotypes (eCOMPAGT) deriving from genetic epidemiological studies. eCOMPAGT securely stores and manages genotype and phenotype data and enables different user modes with different rights. Special attention was drawn on the import of data deriving from TaqMan and SNPlex genotyping assays. However, the database solution is adjustable to other genotyping systems by programming additional interfaces. Further important features are the scalability of the database and an export interface to statistical software.

**Conclusion:**

eCOMPAGT can store, administer and connect phenotype data with all kinds of genotype data and is available as a downloadable version at .

## Background

Genome-wide association (GWA) studies are a very successful approach to identify the genetic basis of complex human diseases. In fact, more genetic risk factors for common diseases were identified in 2007 and 2008 than had been collectively reported before [1-3]. GWA studies are based on the concept that by testing a large number of single nucleotide polymorphisms (SNPs) and combining the SNP-data with phenotypical data allows detecting disease-associated alleles. The recent explosion of genetic discoveries is based on cost-effective genotyping technologies that can assay hundreds of thousands of SNPs simultaneously and the statistical power of GWA studies, which normally include study populations of several thousand individuals. These advances have allowed a systematic, even 'agnostic' approach to genome-wide interrogation. This hypothesis-free unbiased method is *the *major advantage of GWA studies and allows the identification of new disease pathways which would be hard to discover otherwise.

However, as reports on questionable genotype-phenotype associations are increasing [4,5], the key to separate true associations from the blizzard of false positives are either well powered studies with thousands of participants and/or replication of the initial GWA findings in further populations. The purpose of a replication study is to evaluate a positive finding from a previous study, to provide credibility that the initial finding is valid. Replication is essential for establishing the credibility of a genotype-phenotype association, whether derived from candidate-gene or GWA studies [6].

The immense data generated in GWA and replication studies impose new demands on data management systems. While large genotyping centres normally possess their own bioinformatics division for data management, smaller laboratories performing replication and genetic association studies have to face the administration of rapidly increasing data volumes both from genotyping as well as phenotyping procedures from different study populations.

Some commercially available products allow the management of genetic and phenotypic data (for a review see [7]) and one freely available sophisticated tool for handling genotype and phenotype data was presented by Fiddy and colleagues [7]. The paper at hand presents a database system for an "efficient COmbination and Management of Phenotypes And GenoTypes" (eCOMPAGT) deriving from genetic epidemiological studies. eCOMPAGT securely stores and manages genotype and phenotype data and enables different user modes with different rights. This is especially important in the context of clinical studies, where patient-relevant information is being stored, but should not be shared with statisticians or lab technicians. The implementation of data deriving from the SNPlex system (Applied Biosystems, Foster City, USA) is one important feature. The SNPlex Genotyping System enables the simultaneous genotyping of up to 48 SNPs in one run against a single biological sample. A specific collection of 48 SNPs is termed "SNPlex pool". This system is ideal for fine mapping and candidate gene analysis as well as replication studies, and is being increasingly used in genetic epidemiological laboratories world-wide. However, the database solution is adjustable to the other genotyping systems by programming additional interfaces. Further important features are the scalability of the database and an export interface to statistic programs.

## Construction and content

**Figure 1:**
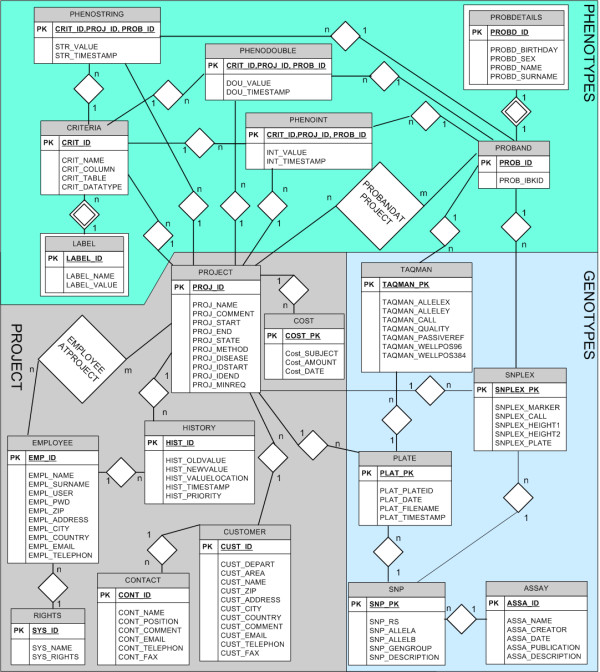
**Final E/R diagram of eCOMPAGT**. Entities are represented as rectangles and relationships as diamonds. For example, the relationship PLATE – SNP means that a SNP consists of one or more plates, and a PLATE is assigned to one SNP.

**Figure 2:**
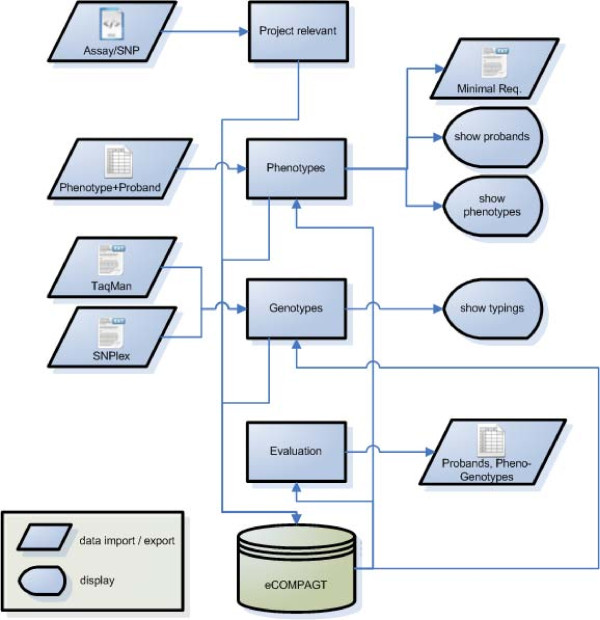
**Flow chart of data import and export**. eCOMPAGT stores phenotypes and genotypes in an efficient way and supports a combined export (rhomboids). The GUI represents the imported data in 4 menu items (rectangles) and offers an overview of the different type of data.

**Figure 3:**
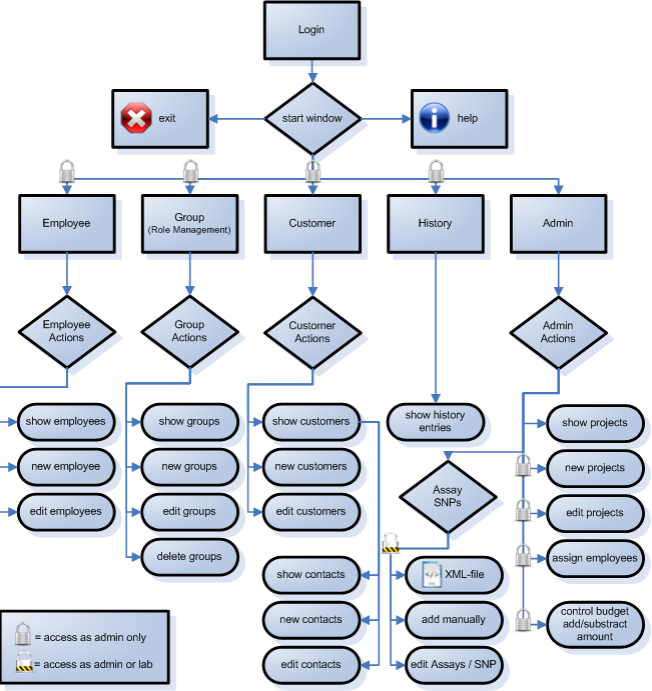
**Flow chart of the Customer Relationship Management system**. eCOMPAGT contains typical elements of a CRM-system like management of team members and customers, an integrated role management, a history (versioning of modified values) and additional administrator tasks.

eCOMPAGT is a Laboratory Information Management System (LIMS) to store the complete set of project-relevant information in one single database schema in a compact, efficient and straightforward way (Figure 1):

**Phenotypes**, which often differ from project to project in their formalization, coding, data format and number of positions after the decimal point, can be imported from a variety of different input files (as Excel File Format BIFF8) into the database. This import can be done in a single step, independent of the number of stored phenotypes in the original file. Approved **genotypes **of different genotyping methods (e.g. TaqMan, SNPlex) can be stored efficiently in order to enable a later combination of selected phenotypes and selected genotypes. **SNP information **from TaqMan or SNPlex assays can either be extracted automatically from XML-files or be added manually to eCOMPAGT (Figure 2). Elements of a classical Customer Relationship Management System, such as a structured **customer **and **project management**, are part of the system (Figure 3). eCOMPAGT offers a **role-management **for coworkers and customers and controls the access to proband-specific information in that way. Additionally, a unique database account for each user is created to increase safety and access proband-specific information only with appropriate rights. The **export interface **enables a combined export of selected phenotypes with corresponding genotypes to common statistical software. Discrete phenotypes can automatically be recoded into numbers. Furthermore a **versioning capability **is used for keeping track of incrementally different values and of project-specific information ("history").

### Detailed Features

#### Phenotypes

Information of phenotypes and subject-related information (e.g. name, gender) are normally linked in one single BIFF-file; for continuous storage, this file needs to split into sensitive subject-related information and the corresponding phenotypes. A major advantage of working with BIFF-files is the usage of the JExcelAPI [8], which facilitates the read-out and read-in. Without that, the import of phenotype and proband information and the separation in different relations with automatic data type determination in a reasonable amount of time could not be handled in a feasible way. During the read-in, column headings, proband information (can be easily selected by the user) and phenotypes are displayed and further inserted in different relations. Phenotypes are stored in our database using the "generic triple", which consists of a data-value, a criterion (= subject) and a data type. At the read-in the data type is determined and is stored together with the header of the column in the table CRITERIA. Furthermore the actual values of the columns are stored in one of three relations (PHENOINT, PHENODOUBLE and PHENOSTRING). Before storing, the user gets an overview of all column types, and can alter possible clerical errors. For example, if a column exists of 1000 integer values and 1 string value, the whole column would be stored in the PHENOSTRING relation). Problems like clerical errors or special characters (like '/') can never be avoided, which make the PHENOSTRING relation absolutely essential. The separation of the file ("generic triple") leads on the one hand to a more efficient database schema and on the other hand to the important feature of information privacy. Proband-specific information is only visible if the role management of the application and the database internal rights (insured via a unique database account) are sufficient. This provides the opportunity to share sensitive data while at the same time a protection of personally identifiable information is guaranteed. With this schema an import of 1 million phenotype values (10.000 probands, 100 phenotypes) is possible within proper time (please see below under "Performance"). Due to memory saving capabilities the phenotype values are stored in three different tables, according to their data types.

#### Genotypes, Assays, SNPs

Calls, generated by different genotyping methods, are stored by parsing tabulator separated files [9]. Additional data like well position, marker name, call position and quality value are inserted into the schema. A metadata relation (PLATE) assists in avoiding the storage of redundant data and adds additional information without further effort. In a first step, the delivered XML data with information about the TaqMan and SNPlex genotyping assays has to be imported into eCOMPAGT. This step is usually performed automatically, but a mask for manual import is available as well (for Applied Biosystems 7900 HT Fast Real-Time PCR System). Following the definition of TaqMan genotyping assays or SNPlex pools, the genotype calls can be imported, which are linked to phenotypes by an included subject identifier.

#### Export

After the read-in of phenotypes, their values and subject-related information, lab technicians and statisticians need access to different kinds of information. This is a critical aspect and a user with administration rights (i.e. the lab head) is responsible to release adequate and sufficient information for the specific downstream application. As the released data can be modified at a later time, a dynamic and flexible flow of information is guaranteed. Apart from releasing selected information for laboratory applications, a second kind of data export is required to merge phenotype with corresponding genotype information for statistical data analysis. This aspect is critical and only accomplishable with appropriate rights. The combination of different types of information can be done with an intuitive graphical export interface. As a special feature, string data types are recognized automatically and can be labelled with integer values in order to enable an easy export to various statistical packages.

#### Project Management

An integrated project management was revealed for storing genotypes, phenotypes and further information. Typical elements of a CRM (Customer Relationship Management) system, such as the storage of customers, team members and project information, are handled by eCOMPAGT. Each academic and commercial institution wishes to have an overview on how much work on each project is already done and in how many scientific cooperations they are involved. eCOMPAGT can provide statistics on these issues. Furthermore, a flexible assignment of selected team members to each project helps to prevent unauthorized access. Another important feature is the role and rights management, which enables the assignment of different rights to each team member. The role of an employee is defined once and stays the same over all projects (e.g. lab technician). However, the rights of the employees are adjustable, meaning that a co-worker can access only projects to which he or she is assigned. To even more increase security aspects, we extended the design by the use of several database users, assigning each eCOMPAGT user a separated database access. With this feature eCOMPAGT enables both a virtual private databases feature of Oracle 8i (and onwards) and data hiding via database views, which guarantees that each user can access only the data he has been authorized for and all security-critical data is locked.

We are aware that regulations on a national or institutional basis require a clear separation of the main data from personal identifiers. This would simply be possible by using a unique ID which is used in the main data base as identifier and which is "connected" to the person-identifying data only on a separate PC which is not connected to the network and which can only be accessed by authorized persons.

Another part of our project management is the **administration of project-related financial resources**. These resources can be monitored and administered by eCOMPAGT. This helps to get an overview of the current budget situation and enhances controlling and reasonable handling of financial resources. The budget management also allows having a continuous overview on the project and alarms when the costs of the project get out of control.

#### Versioning

A versioning function enables editing and erasing of phenotype values and genotype calls and even a complete removal of subject information if necessary (with proper rights). This feature enables to keep track of who changed or deleted any piece of information stored in the system. As obsolete values are still held in the database, it is possible to undo any changes at every time.

## Utility

**Figure 4:**
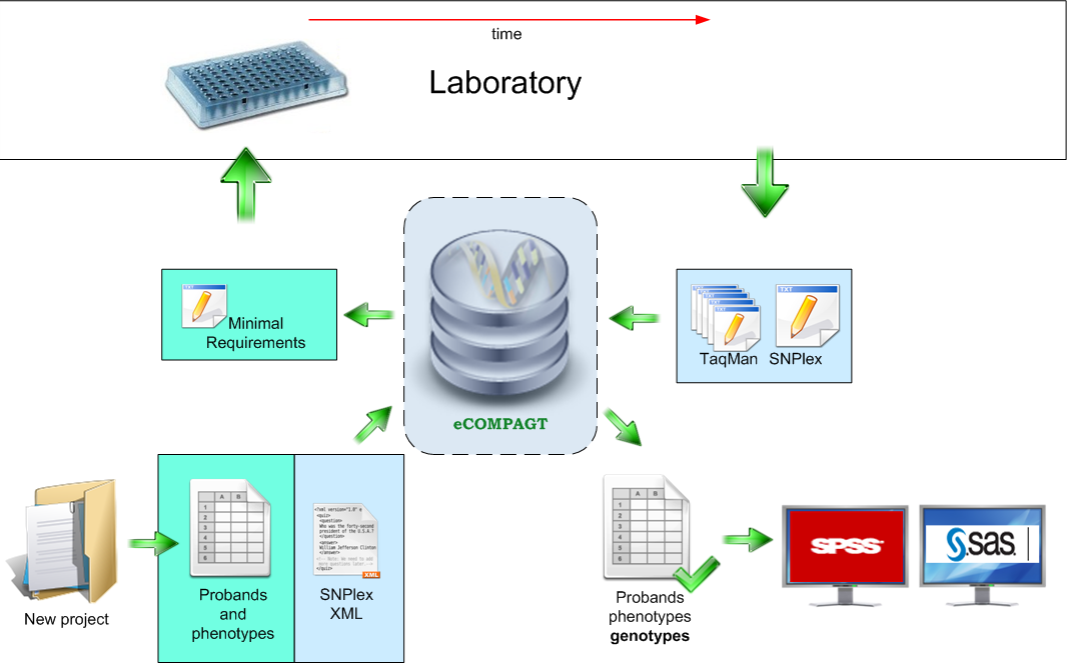
**Workflow of eCOMPAGT**. eCOMPAGT simplifies the workflow and supports a more flexible interaction between laboratory and administrative tasks. eCOMPAGT delivers minimal requirements to the laboratory and receives final and reviewed genotypes.

The main tasks of a centralized organisation of biological data are to avoid errors in merging genotypes and phenotypes, to guarantee information privacy and to handle a huge amount of data in straightforward fashion. Due to the fact that an intervention with an existing workflow process is critical, the designed application has to be intuitively useable and accepted by the end users. The laboratory receives the delivered *minimal requirements *for further processing, which is illustrated in Figure 4. Minimal requirements are unambiguous identifiers of subjects, stored in a simple text file, which guarantee a correct mapping to the corresponding DNA material. The ideal case is that only the internal ID (generated by eCOMPAGT) has to be released as a minimal requirement, but experience shows that usually additional information (e.g. the gender of the subjects) is required to guarantee a correct identification of samples for genotyping or control purposes. Thus, the option for e.g. the lab head to alter the number and types of the minimal requirements is essential. It is worth noting that a stringent quality control has to be performed in the laboratory (testing for Hardy-Weinberg-Equilibrium, 10% quality controls, no template controls, ...) so that only final and reviewed genotypes are stored in eCOMPAGT.

### Graphical User Interface (GUI)

An intuitive user interface was designed with Java Swing [10], extended by Java SwingX components [11] and improved with Substance [12].

**Figure 5:**
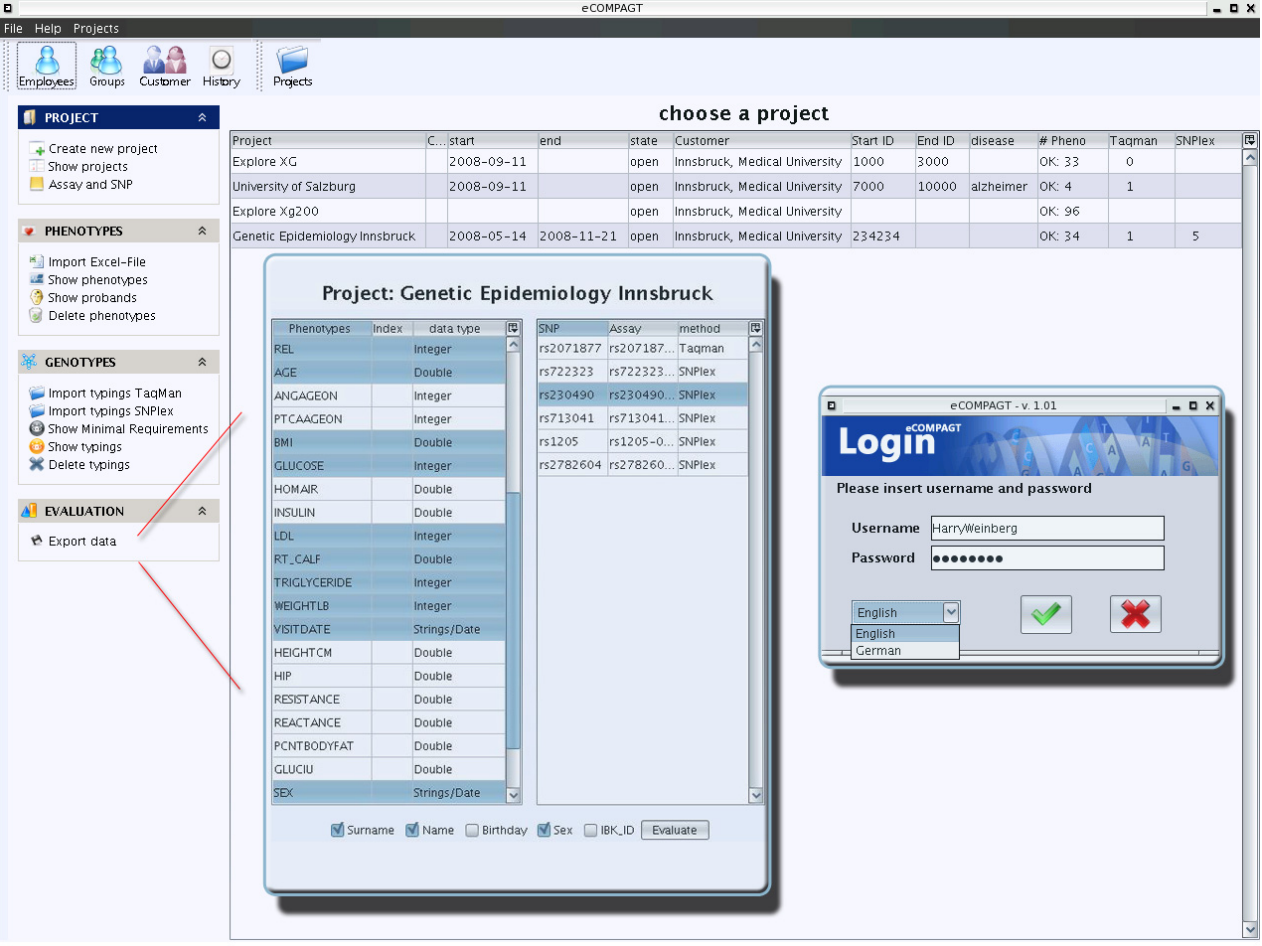
**The main menu items Project, Phenotypes, Genotypes and Evaluation appear after a successful login with proper rights. eCOMPAGT supports an intuitive usable export interface**.

The GUI is separated into two parts: the first part encompasses elements of a typical CRM system. Customers, team members and projects are addable, editable and erasable by dedicated menu items (Figure 5).

Figure 5 shows the second important part of eCOMPAGT, where the import and export of genotypes and phenotypes is performed ("Evaluation"). During the import of phenotypes and subject information, an internal identifier ("minimal requirement") has to be assigned to each subject. This can be achieved automatically or if a unique identifier is already available by highlighting the according column in the GUI. This informs the application to use the highlighted column as a unique identifier that makes a later combination possible and ensures subject privacy.

### Implementation

The amount of data, which is derived from different genotyping methods, is steadily increasing and high-performance queries based upon an efficient data model are required. The entity-relationship modelling was the first step to produce a conceptual data model and we observed that the normalization model fits best to our requirements.

Figure 1 shows our final database structure, in form of an Entity-relationship model (Chen notation).

eCOMPAGT was originally designed for IBM DB2 Version 9. However, it can also be used with Oracle Database 10 g. With the assistance of Hibernate [13], an object-relational mapping tool (ORM), the storage of Java objects in a relational database is possible. eCOMPAGT uses this feature to guarantee an abstraction to the underlying database layer. Starting with Version 5, JDK (Java Development Kit [14]) offers the usage of typesafe annotations. The JPA (Java Persistence API, [15]) uses this feature and defines the mapping syntax, the semantics and the life cycles of objects and query possibilities, which are accessible by the Hibernate Entity Manager. With JPA all configuration files of eCOMPAGT are held in one file (*persistence.xml*) and the bindings to the database are directly included in the source code (via annotations, [16]). This eliminates the need of XML mapping files and simplifies the configuration.

We tested this portability by using different relational databases (DB2, MySQL). After small adjustments of the configuration file and the schema (syntax of sequences), eCOMPAGT was applicable with both database systems. Additionally, the application runs on Linux and Windows systems given the availability of a Java Virtual Machine (part of Java Runtime Environment).

To guarantee a fast import/export of data and optimized queries, we applied JDBC (Java Database Connectivity [17]) connections and index optimization.

### Performance and scalability

The application was designed for the use in the intranet of a research institution and constitutes a rich client, which depends on a centralized database. Additional indices were generated to optimize the queries within the database. As a consequence, the upload of phenotype or genotype files requires more time than without additional indexing, but with the benefit of fast query results. We tested the system with 20 different projects, each containing 10,000 samples. For example the query of 10,000 samples of one project takes less than 1 second. For time-critical queries stored procedures were additionally used. As a database server we used two different machines: an Intel Pentium 4 Server with 2.8 GHz and 786 MB RAM in the beginning and moved to a server with 8 processors (quad core) with 2.6 GHz and 16 GB RAM later on.

When talking about scalability, two important features have to be addressed: time complexity and space complexity. Concerning time complexity, eCOMPAGT is based upon a relational database system, as Oracle or DB2. Hence time scalability is given directly by the system: using the B-tree for indexing, simple algorithms for search-, insertion- and deletion-functions of data records are provided in *O(logm(n)) *time.

Concerning space complexity, the relational databases offer several possibilities for huge amount of data, like cluster architectures, in order to avoid issues regarding limited storage requirements.

### Comparison to other systems

At the time of publication, we are aware of two other systems that are similar to eCOMPAGT: IGS [7] and SNPLims [18]. In contrast to IGS and SNPLims, which have a web-based interface and command-line clients, the interface of eCOMPAGT is a user-friendly Java-based client. A major advantage of eCOMPAGT is its platform independency, whereas SNPLims requires a Debian server, and IGS was developed for Windows systems. The database system for eCOMPAGT is Oracle, for IGS it is MS SQL, and for SNPLims it is PostgreSQL. Especially in the upload of phenotypes, the three systems differ dramatically. While eCOMPAGT offers an easy upload through BIFF files, IGS requires a full definition of phenotypes before the upload, and SNPLims separates phenotypes from demographic data. IGS and SNPLims are designed for handling high-throughput genotyping data deriving from platforms like Illumina, MegaBace and Sequenom; eCOMPAGT is a solution for small to medium-sized laboratories which use TaqMan and SNPlex for genotyping. An extension to the import of STRs will be available in the next version of eCOMPAGT. A nice add-on of eCOMPAGT is the availability of a history function, which enables an easy and reliable tracking of data modifications.

Other projects that were similar by concept but that would not meet our special requirements were SNPP and PACLIMS. SNPP [19] is an open source application based on MS Access or on a MySQL database. The application works well with genome data files in the Invader Analyzer (Third Wave Technologies, Madison, WI) format and phenotypes are accepted in Excel format. Neither project management nor history functionality for controlling data modification are available. Furthermore multiplex genotyping methods are not supported. The laboratory information management system PACLIMS [20] was developed for Unix/Linux systems with a PostgreSQL database. It can be used as a model for high throughput mutational endeavours.

A tool which is not in competition to our system, but could be a very valuable complementation to eCOMPAGT, is TIMS (TaqMan Information Management System) [21]. TIMS is a package of Visual Basic programs focused on sample management and on the parsing of input and output TaqMan files and is thus a great tool to organize the data flow within the genotyping laboratory, before entering the results into eCOMPAGT.

## Discussion

eCOMPAGT provides an easy usable software application for researchers without background in the area of computer science. Phenotype files can be imported into the database at once and no further information about data types of columns or the position of specific phenotypes in a file has to be known in advance. Data types are determined at run time and are stored in appropriate relations. This can be seen as a major advantage of eCOMPAGT compared to IGS [7], in which a predefined format must be maintained and the upload cannot be done in one step. It is even possible with eCOMPAGT to export genotype and phenotype information in one single file. By defining minimal requirements, the amount of necessary information that is being handed over to the laboratory is alterable for each project. Even genotypes generated in the laboratory (from TaqMan and SNPlex assays) are imported without any transformation or code conversion, which reduces the error rate dramatically and constitutes a significant advantage of eCOMPAGT. In addition, the ability to also administer and monitor the financial resources of a certain research project renders this tool as an all-in-one-solution for project management in a genetic epidemiological research laboratory.

At the moment, the import of phenotypes can exclusively be done via the use of EXCEL spreadsheets; tab-delimited files need to be converted into EXCEL files before. We decided to work with EXCEL sheets as the exchange of phenotypes and proband-related data between institutes is in most cases being done via EXCEL.

Although there are already similar software solutions available that would partially fulfil our requirements, we decided to create our own open-source software solution in order to be highly flexible when new genotyping methods are being established in a laboratory, for example SNPlex or MLGA [22], a rapid and cost-efficient assay for gene copy-number analysis.

The idea of eCOMPAGT and its dynamic approach makes it a powerful tool for even small laboratories and could lead to a higher consumer acceptance of genetic analyses without any bioinformatic support.

### Future perspective

With the increasing appreciation of the importance of copy number variations, a recently discovered form of genetic variation that involves deletions and duplications of DNA fragments in the size of 1 kb to several megabases [23], the storage of results from copy-number detecting algorithms is envisioned to be enabled in a future version of eCOMPAGT. In advance, as a smaller form of genetic length variability, we will implement the import, storage and export of short tandem repeats (STRs) into the next version of eCOMPAGT.

## Conclusion

eCOMPAGT has been developed to facilitate replication studies of genotype-phenotype associations in the field of genetic epidemiology. It enables the handling of large volumes of genotype and phenotype data in a secure manner by assigning different rights to different user types. A detailed versioning enables tracking and monitoring of all data manipulations and thus guarantees traceability of all changes and edits. Although it is not the first tool that permits a combined management of phenotypic and genotypic data, it is the first tool that allows also the import of SNPlex data and a control over the financial resources of different research projects and which might fit more the needs of small- to medium-sized genotyping laboratories. The flexible structure of the database scheme enables a straightforward implementation of relations and the adaptation of the source code for the storage of new genetic variants such as copy number variations.

## Availability and requirements

Project name: eCOMPAGT

Project home page: 

Operating system(s): Platform independent

Programming language: Java

Other requirements: Java 1.6., relational database (tested with Oracle Version 10 g)

License: GNU GPL

A user manual for eCOMPAGT can be downloaded from 

## Authors' contributions

SS and HW were responsible for programming and designing of eCOMPAGT and drafted the manuscript. FK and GS initialized the project and edited the manuscript. SC tested the software for functionality in the lab. AB supervised the project and drafted the manuscript. All authors read and approved the final manuscript.
